# Personalities, Preferences and Practicalities: Educating Nurses in Wound Sepsis in the British Hospital, 1870–1920

**DOI:** 10.1093/shm/hkx016

**Published:** 2017-05-04

**Authors:** Claire L Jones, Marguerite Dupree, Iain Hutchison, Susan Gardiner, Anne Marie Rafferty

**Keywords:** nursing, education, asepsis, antisepsis, bacteriology, surgery

## Abstract

The history of nursing education has often been portrayed as the subordination of nursing to medicine. Yet, as scholars are increasingly acknowledging, the professional boundaries between medicine and nursing were fluid in the nineteenth and early twentieth centuries, when both scientific knowledge and systems of nurse training were in flux. Through its focus on the role of medical practitioners in educating nurses in wound sepsis at four British hospitals between 1870 and 1920, this article attempts to further unite histories of medicine and nursing. It demonstrates that, in this period of uncertainty, the ideas and practices relating to antisepsis, asepsis and bacteriology disseminated to nursing probationers depended on the individual instructor. In demonstrating the localised nature of nursing education, this article argues that further analyses of clinical problems like wound sepsis may enable historians to more clearly identify the importance of professional collaboration within the hospital.

It is as desirable that the nurse should know something of medicine as that the medical man should know something of nursing, and that there should be mutual respect and loyalty. … Medical men, or at any rate some of them, take an important share in the work of the teaching [of nurses].^1^

The history of nursing education has often been portrayed as the history of the subordination of nursing to medicine, of separate gendered professional spheres, with little negotiation in the power relationship between them.^2^ Despite attempts to bring together narratives of Victorian male doctors and female nurses, professional boundaries between the two are often presented as fixed in some fashion.^3^ Significant differences and inequality between the two undoubtedly prevailed, but this article presents a more complex and nuanced picture: one in which both relationships and knowledge were in flux in the late nineteenth and early twentieth centuries, with greater credence given to those who advocated mutual respect and reciprocity in understanding each other’s craft. Indeed, in 1878, John S. Bristowe, physician at St Thomas’ Hospital and nursing instructor at the Hospital’s Nightingale School for Nurses, wrote an essay from which the above extract is drawn entitled ‘How far should our hospitals be training schools for nurses?’. Bristowe’s views about the importance of involving hospital doctors in the training of nurses were clear: alongside the practical training nursing probationers undertook on the wards, medical men ‘can teach nurses something of what they know. … I refer to the principles of medicine and surgery and to clinical instruction in the wards’. According to Bristowe, in no area of medicine or surgery was the inclusion of medical instruction in nurse education more important than in the principles and practices related to preventing and controlling wound sepsis: antisepsis and asepsis. Thus, in the same year as the publication of his essay, Bristowe included these principles and practices in his lecture entitled ‘germ theory’ to the thirty probationers enrolled at the School.^4^

This article aims to unite the professions of medicine and nursing by exploring the ways in which medical men attempted to embed antisepsis and asepsis into the nursing curricula at British hospitals between 1870 and 1920. What follows places these attempts in a wider context of debates over both the changing scientific knowledge and practices of wound sepsis, and the form and content of nurse education; it explores the kinds of knowledge that hospital medical staff at institutions which had new nurse training schools in this period were willing and able to disseminate to nursing probationers. It not only addresses the taught content, but also highlights the preferences of the instructors, what shaped their preferences and what processes they used to disseminate this knowledge. The dissemination of wound sepsis knowledge from physicians and surgeons to nurses, and the corresponding transfer of this knowledge into practice were far from simple processes. With a lack of national consensus, at least until the Nursing Registration Act of 1919, each instructor within each hospital set his or her own nursing course syllabus and forms of assessment. Moreover, debates within the medical profession over wound sepsis pathology complicated such translations. This article’s focus on wound sepsis education for nurses then provides new insights into the relationship between nursing education and medical theory and practice, and into some of the underlying politics and disciplinary boundaries between the professions of medicine and nursing in the late nineteenth and early twentieth centuries. We argue that wound sepsis was part of a ‘negotiated order’ of interaction between nurses and doctors, which emerged from local ecologies of practice at a time when the details of bacteriology and their implications for practice were in flux. At the heart of this analysis then is the relationship between medical men and nurses and where the boundaries of their respective professions lay. As we shall see, the direction of dissemination was not top-down one way traffic from doctor to nurse but more dynamic in nature relying upon the negotiation between doctors and nurses at local level, each bringing a different perspective and contribution to theory and practice.

Why was medical instruction for nurses especially important for combatting wound sepsis? First, high rates of wound sepsis and ‘hospital’ diseases or ‘hospitalism’ from the 1860s threatened to make hospitals places of fear, jeopardising the standing and reputation of the institutions, their associated physicians and surgeons, and the expansion of their medical schools.^5^ New pathological understandings of wound sepsis and methods to prevent and treat it, together with the hygienic preventive practices conducted by the nurse, had become pivotal. Secondly, medical men were only ever intermittently present on wards; they relied upon nurses to carry out their instructions, acting as the eyes and ears of the doctors in their absence.^6^ Nurses therefore needed to be taught both how to carry out the instructions, and to appreciate the ‘why’ as well as the ‘what’ of what they were doing, in order for medical and surgical treatment to be successful. Thus, the standing of a hospital and professional reputations relied heavily upon the prevention or treatment of wound sepsis and other hospital-acquired diseases, which in turn depended on the quality of nursing: as a result, nursing became an important focal point for reform and training.

Yet, despite the importance of educating nurses in wound sepsis, existing histories tell us little about what nursing probationers were taught, who taught them or the ways in which this was perceived to affect hospital practice. The dearth in scholarly work on this topic may result from the fact that there was a general lack of consensus in the late nineteenth and early twentieth centuries on the nature, content and format of the taught curriculum at the new nursing training schools attached to large teaching hospitals. Equally, there was little agreement surrounding wound sepsis pathology and the practices of antisepsis and asepsis.^7^ Indeed, to date, only Alison Bashford and Pamela Wood have begun to treat debates over nursing education and over wound sepsis as intricately intertwined subjects.^8^ While acknowledging the impact of socially-constructed prejudices resulting from gendered professional hierarchies outlined in much of the historical literature, Bashford and Wood provide a more nuanced picture of the relationship between nursing and medicine and, accordingly, between intellectual content and morally embedded practical work. Indeed, while histories of nursing education have tended to follow Florence Nightingale’s vision of the ideal nurse as the inculcation of moral virtues within practical ward work as the antithesis of intellectualism resulting from taught instruction, Bashford’s chapter on late nineteenth-century germs and the gendered practitioner and Wood’s survey of the role of hospital nurses in preventing and treating wound sepsis in Britain, Australia and New Zealand between 1895 and 1935 highlight the importance of medical instruction to nurses; their research identifies the different motivations of medical men in instructing nurses, as well as some of the methods used to teach them. Anne Hanley’s study of the training and practice of midwives between 1895 and 1914 provides similar insights into the teaching about *Ophthalmia Neonatorum* to midwives.^9^ There were certainly deep anti-intellectual prejudices attached to nursing, as part of a broader late nineteenth- and early twentieth-century prejudice against women’s work in general, the education of women, and the feminisation of skill.^10^ Accordingly, histories of nursing education have perceived a general lack of sympathy from doctors towards intellectual improvements in nurse education, which doctors justified by differences in male and female physiology, the polemical private and public spheres, and the desire to protect the boundaries of professional medicine.

But there were also very real nineteenth-century attempts to reform nursing along the lines of medicine, spearheaded by Mrs Bedford Fenwick and advocated by some physicians and surgeons, which have yet to be assessed in detail.^11^ The ‘better educated’ nurse was regarded as a more reliable assistant, capable of following instruction more faithfully because she would understand the rationale for practice. This was an important motivator for doctors supporting educational reform in nursing more generally.^12^ Bashford argues that some doctors were able to accept the dissemination of medical knowledge because it was repackaged into established forms of nursing knowledge about cleanliness and hygiene, while Wood highlights the ways in which different surgeons saw the intellectually educated nurse as either supporting or sabotaging his practice. Hanley demonstrates that the integration of bacteriological causation for *Ophthalmia Neonatorum* into midwifery education was heavily dependent upon the enthusiasm of individual teachers and their conception of what constituted suitable knowledge for midwives.

Variations in practices and the preferences of individual teachers in the dissemination of knowledge over wound sepsis and its pathology were also evident in medical education. Michael Worboys, and other representatives of the practical turn in the history of medicine, have identified the wide-ranging responses among British surgeons to antisepsis, asepsis and bacteriology, the emerging science dedicated to the study of micro-organisms including those found in wounds.^13^ British surgeons’ adaptations of principles and practices meant that instruction on these topics varied across individual hospitals and medical schools.^14^ London hospitals, for example, were well known for their initial hostility towards Lister’s antiseptic ideas and techniques. The influence of Lister’s teaching on a whole generation of medical students in Glasgow and Edinburgh in the late nineteenth century facilitated the spread of his ideas, and part of his motivation to move from Edinburgh to King’s College London in 1877 was to extend the reach of his influence.^15^ Similarly, by teaching nurses, the physicians and surgeons could enrol large numbers of nurses in their methods and practices, establishing those practices and extending their influence and reputations within and beyond the walls of the institution.

While previous studies have drawn on textbooks as technical manuals that demarcate knowledge between doctors and nurses, this article will also examine other forms of pedagogical tools, including syllabi, teachers’ and students’ lecture notes, and examination papers. In particular, it utilises archival sources from the nurse training schools of four key British teaching hospitals: King’s College (KCH) and St Thomas’ in London, and the Royal Infirmaries in Edinburgh and Glasgow (RIE and GRI). Not only were they among the teaching hospitals most closely associated with the pioneering infection control work of Nightingale and Lister, but, as home to the earliest established and most highly regarded training schools for nurses in the world, they were prominent in debates over the form and content of nursing education in this period.^16^ Moreover, the circulation of leaders between these hospitals, as well as the pre-eminence of these hospitals in both the development of practices for controlling wound sepsis and nursing education, meant that all four hospitals were closely linked. Lister, for example, worked in Edinburgh, before developing his ideas and practices of antiseptic surgery first as Regius Professor of Surgery at Glasgow University from 1860 to 1869, then at Edinburgh University as the Chair of Clinical Surgery, before finally taking his system to King’s College London in 1877 as Professor of Surgery. The early matrons of both the GRI and the RIE trained at the Nightingale School at St Thomas’ Hospital in London. The focus here on four case study hospitals therefore provides insights into the circulation of ideas, people and training methods on the ground.

Further insights are gleaned when analysis goes beyond the mere content of material to focus on form and layout. Indeed, as historians of the book and of science, technology and medicine are becoming increasingly aware, the intellectual content of a particular publication provides only part of the process of knowledge communication. Andrew Warwick’s study of the pedagogy of mathematical physics in Victorian Cambridge demonstrates that knowledge is embedded in the practical skills and technologies, so that forms and methods of communication within a learning environment shaped the messages students received from taught instruction.^17^ By taking seriously both the taught ideas about wound sepsis and the ways in which these ideas were communicated to probationer nurses, it becomes even clearer that ideas were not shaped by some eternal ‘scientific truth’ but by the ‘truths’ hospital medical and surgical staff and associated tutors chose to accept.^18^ In what follows, we chronologically trace changes to wound sepsis content in nursing education at four British hospitals to assess disinfectants and antisepsis in the 1870s; antisepsis, asepsis and the limits of bacteriology in the 1880s and1890s; and bacteriology between 1900 and 1920.

## Embedding Antisepsis into Hygienic Practice at St Thomas’ and the Royal Infirmary of Edinburgh in the 1870s

In 1867, when Rebecca Strong undertook her year-long training at the recently established Nightingale Training School for Nurses with around 15 other probationers, under the watchful eye of Mrs Wardroper, Florence Nightingale’s chosen Matron, her training consisted of ‘kindness, watchfulness, cleanliness … [from] a few stray lectures … and a dummy on which to practise bandaging’.^19^ Yet, within a few years, the tripartite structure of nursing education, including ward work, classes with a home sister and medical lectures by hospital staff became firmly embedded into training schools, first at St Thomas’ and then at the RIE in the mid-1870s under the lady superintendent, Elizabeth Ann Barclay, a former Nightingale. The principles and practices of cleanliness formed an important part of each of the three sections of nursing education, and they also became integral to formalised programmes of lectures in anatomy, surgical nursing and clinical medicine along with the principles of hygiene, at least for ‘special probationers’.^20^ From the 1870s, these principles included a new emphasis on ways in which to treat different types of wounds, abscesses, boils, carbuncles and the four main septic diseases (erysipelas, pyaemia, septicaemia and gangrene) by chemical disinfection. Delivered by the hospital medical and surgical staff and assessed by examinations, these lecture courses represented an expectation among ambitious instructors that probationers (around 50 at the Nightingale School and around 30 at the RIE in 1878) could and should be provided with the underlying intellectual content behind practices to prevent and treat wound sepsis and that this content should be delivered by methods similar to those used to instruct medical students.^21^

At St Thomas’ Hospital in 1873 and at the same time as Lister’s ideas about antiseptic wound treatment were gaining support from some surgeons in the Hospital, John Croft (1833–1905), a surgeon and lecturer in surgery, developed the Nightingale School’s lecture syllabus for surgical nursing.^22^ The lecture course embedded new principles of surgical cleanliness and hygiene into at least seven out of 22 lectures. Lecture topics included the management of wound sepsis (including dressings), pre- and post-operative preparation of patients, bandaging and methods of treatments for ‘hospital diseases’. He informed nurses of surgeons’ practice of dividing wounds into five distinct kinds: incised (a clean cut), lacerated and contused (torn and bruised cuts resulting from blunt instruments), punctured (from a stab), and poisoned (a dirty wound).^23^ His inclusion of lectures about the prevention and treatment of erysipelas were particularly pertinent, given the large number of outbreaks in the hospital wards during the 1870s and the role of nurses in managing these outbreaks.^24^ Croft’s lecture syllabus, distributed to probationers with whom the hospital was in written communication, was an early prospectus and enabled the Nightingale School to promote the layout of the course to potential, as well as existing, probationers.

In 1875, two years after Croft began to deliver his lectures at St Thomas’, surgeon Joseph Bell (1837–1911) began to deliver a series of lectures to nurses at the RIE.^25^ Published for the first time in 1887 as *Notes on Surgery for Nurses* and into its sixth edition by 1906, the book contained chapters on the clinical signs of inflammation, suppuration, ulceration, gangrene, pyaemia, septicaemia, and on the healing of wounds by first, second and third intention.

Crucially, the content of both Croft and Bell’s lectures not only represented their personal views about wound sepsis, but also their understandings of the nature of nursing knowledge and the relationship of this knowledge to practice. Unlike Nightingale and others, who maintained that nursing education should be entirely distinct from medical education in both content and format, Croft’s and Bell’s simple willingness to instruct probationers reflects their view that the contributions of medical men were an important way to improve nursing practice and in turn, surgical practice. Their prioritisation of ward, wound and surgical cleanliness, indicated by their delivery of lectures on these topics before any others, suggests they placed a high value on nurses learning about wound sepsis in order to embed the appropriate techniques into their future ward and operating theatre practice.^26^ In particular, one of Croft’s lectures titled ‘Disinfectants and Antiseptics’ stated:

I shall have talked to you of disinfectants and antiseptics to little purpose if I have not impressed upon you the great necessity there is for employing these agents. Medical and surgical diseases are spread by the infectious particles and gases carried about by the air or by persons and things too numerous to mention, things ordinary and extraordinary. One should be suspicious of every fluid or material in a sick room or near a sick room which could by any possibility putrefy or decompose, and should render it proof against the infective process by using a suitable preventer of putrefaction. I have not given you detailed instructions how to disinfect every article after every special disease or how to disinfect rooms that have been occupied by the subjects of contagious diseases, or how to perform duties which belong to special sanitary officers as they are called, but I have given you information which should be of service to you in your nursing duties.^27^

The above quotation not only highlights the information Croft thought nurses should know to enhance their work, but demonstrates that he used this lecture as a platform to incorporate Lister’s ideas about antisepsis into the already established nursing practices of cleanliness and hygiene. Croft was one of the first hospital surgeons in London to express enthusiasm for Lister’s ideas and practices, which were based on a germ theory of putrefaction; by the 1890s, he continued Lister and William Macewen’s work on simple fractures.^28^ While Croft does not mention Lister by name, his support for Lister is demonstrated by the inclusion of descriptions of ‘infective particles’ and the ‘infective processes’ of putrefaction and fermentation (terms Lister used for the putrefactive process of sepsis), types of disinfectant (e.g. carbolic acid, permanganate of potash (Condy’s fluid), chlorozone, heat and water) and methods for their use in treatment, together with a discussion of the most up-to-date theories behind these processes, particularly John Tyndall’s ideas about infective organisms. Suggesting that nurses were already familiar with Tyndall’s views on putrefaction, Croft asks:

Do you remember Professor Tyndall’s lecture on ‘Dust and Disease?’ He showed the presence of dust in air which seemed perfectly pure and empty to the unaided eye, and he did it by means of a beam of very strong condensed light. The particles or germs exist in the atmosphere, though to our naked eyes the air is empty and invisible. Now, whatever the hurtful things may be which cause offence or produce disease we want to *destroy*, *neutralise*, or *prevent*.^29^

Croft’s honest revelation here of the uncertainties among medical men during the 1870s about what these infective organisms were—‘whatever the hurtful things may be which cause offence or produce disease’ or in other words, whether the organisms detected in the processes of putrefaction and fermentation were plant- or animal-derived and whether these organisms were generated spontaneously or needed seeds or ‘germs’—highlights his view that nurses should be privy to such debates and were able to understand the potential existence and role of micro-organisms in wound sepsis.

Croft delivered the lecture several times each year to the same cohort (in at least one instance he gave it three times a year so that night nurses could be present), indicating the special importance he placed on the lecture. The subsequent publication of Croft’s lectures as a neat booklet and Bell’s as a textbook suggests they had an audience beyond St Thomas’ and Edinburgh, and that other training schools also prioritised wound sepsis management.

Yet, Croft’s and Bell’s continued emphasis on the importance of ventilation, fresh air and cleanliness, principles of nursing advocated by Nightingale and representative of established practices identified by Bashford, highlights their view that nurses should be taught underlying principles only when they felt it was useful to nursing practice.^30^ Cleanliness, Croft argued, should be prioritised and disinfectants should only be resorted to once these elements have been attended to, while Bell emphasised the practical removal of dirt and the regular changing of dressings.^31^ Bell argued that hospital gangrene, pyaemia and wound sepsis could only be banished from hospitals by the practice of ‘real surgical cleanliness’, which included the ventilation of wards and the cleanliness of floors, walls, dressings and bedlinen, alongside the ‘most exact washing of the wound’ after operations with a 1–1000 solution of bi-chloride of mercury.^32^ Like Lister, Bell experimented with antiseptic agents, and by 1887, saw bi-chloride of mercury as a viable alternative to carbolic acid.^33^ Following in Lister’s footsteps may have resulted from his close relationship with James Syme (1799–1870), who embraced antisepsis towards the end of his life and was Lister’s father-in-law.^34^

Moreover, Croft and Bell’s emphasis on the importance of practical intervention—on what the nurses could observe and actively do, such as wound dressings, handwashing and disinfection—limited the time for instruction about theory.^35^ There is little evidence to support the claim that Croft and Bell shared the views of some practitioners that nursing knowledge should be limited in order to maintain professional hierarchies or to prevent overstretching the intellectual capabilities of the nurse. Mary Crossland, a diligent home sister at St Thomas’ between 1874 and 1896 committed to embedding medical knowledge into the nursing curriculum, complained that some doctors seemed to think the nurses did not need to fully understand their subject, but she was impressed that Croft did not conform to this way of thinking.^36^ The limitations Croft and Bell placed on pathology therefore resulted from practical necessity. In addition to saying, ‘I have given you information which should be of service to you in your nursing duties’, Croft argued that ‘whether they [micro-organisms] are the causes of putrefaction or the result of the process, has not important bearing on the matter we have in hand’, while Bell stated that ‘we need not greatly care to discuss what is called germ theory, which some believe in, and some do not’ and ‘it matters little to us what theory is correct, or indeed if any is, if only we remember that the malady we are to describe is found in cases where pus is putrid’.^37^ While it might be tempting to read their views as patronising, their key point—that organisms existed and must be destroyed through disinfection—may also indicate an impatience with the uncertainties in sepsis causation as a distraction from ensuring strict hygiene and cleanliness. Similarly, Bell’s admission that there were uncertainties surrounding the causative nature of wound sepsis accompanied his dismissal of the need for nurses to learn the theoretical underpinning of inflammation and hospital diseases, claiming that theories were ‘too numerous to mention’ within the limited available time for instruction. Moreover, at St Thomas’ by 1878, Croft’s disinfectant lecture was supplemented by Bristowe’s lecture on ‘germ theory’, which presumably included more in-depth discussion of infective organisms. Like Croft, Bristowe was committed to extending nursing knowledge and his firm commitment to germ theories resulted in his delivery of this lecture despite objections from hospital authorities, including Nightingale. Nightingale’s commitment, both to theories of miasma and to the complete distinction between medical and nursing education, provoked her to comment about Bristowe’s lecture: ‘O if they would leave the germs alone & see to the air!’.^38^ Nightingale accepted the existence of germs towards the end of her life, but her practical outlook and her insistence on the highest standards of hospital cleanliness and hygiene were implicit in much of Croft and Bell’s lectures.

Croft and Bell used a mixture of pedagogical approaches and tools to incorporate new knowledge into nurse education, which further highlight their positive assessment of the nurses’ capacity to learn. Diagrams, tables and specimens accompanied Bell’s lectures, which were ‘spoken conversationally’.^39^ Crossland’s notes taken in 1876 reveal that Croft’s lectures were not didactic, but included demonstrations and demanded nurse participation. She describes how Croft conducted lectures in St Thomas’ Alexandria ward to illustrate the evils of putrefaction; he demonstrated antiseptic dressings and demanded that nurses help with the bandaging.^40^ During the first fifteen minutes of his next lecture, Croft asked probationers to explain the evils of putrefaction, in order to ensure that students understood the pathology behind their practices. He also asked one of the nurses to demonstrate the preparation of everything needed for the opening of an abscess and to list the use and purpose of each item.

Moreover, Croft supplemented his lectures and demonstrations with textbooks, recommended to probationers via a reading list. He expected ‘specials’ to undertake self-directed learning during their study period two afternoons a week. While Rafferty and Hanley have argued that textbooks are an important tool for mapping authority relations and for demarcating knowledge and skill between doctors and nurses/midwives, Croft recommended nurses read textbooks written for medical men by medical men and for nurses by nurses, demonstrating his commitment to embedding new principles of antisepsis into existing nursing hygiene rituals.^41^ He recommended Druitt’s highly regarded, medical student staple, *The Surgeon’s Vade Mecum: A Manual of Modern Surgery*, for its chapters on the clinical signs of inflammation and treatments for the ‘common diseases’ of erysipelas and pyaemia, alongside Nightingale’s *Notes on Nursing* and Zepherina Veitch’s *Handbook for Nurses for the Sick*, which emphasised Sir William Fergusson’s principle of cleanliness without antiseptics.^42^ The 10th edition of *The Surgeon’s Vade Mecum* in 1870 only briefly noted the use of carbolic acid in amputations of the thigh, with no discussion of antiseptic theory, nor mention of Lister’s name; however, the 11th edition in 1878 discussed ‘Professor Lister’s beneficent scheme’ in considerable detail in the course of drawing a distinction between proponents of ‘aesthetic’ and ‘surgical’ cleanliness.^43^ Croft recommended that nurses read Nightingale’s *Notes on Nursing*, not just once, but at least four times. Certainly, the lack of specialist textbooks for nurses during the 1870s may have influenced Croft’s choice of books, but his recommendation of textbooks aimed at both doctors and nurses, nonetheless, suggests his expectation that nurses should and could be exposed to medical knowledge.

Not all probationers, however, were prepared for Croft’s method of teaching. For many, it was the first time they were required to study with textbooks, despite having a relatively high level of literacy for female workers at the time. Even ‘special’ probationers, who generally arrived with a better standard of education than ordinary probationers, seemed to struggle. Accordingly, as the second home sister and ‘a more remarkable’ one than the first (Maria Machin), Crossland held supplementary classes.^44^ Crossland’s classes not only highlighted the content of recommended textbooks, but also offered instruction about how to read, ‘otherwise they dip and don’t know what to read’; in addition, she went over the doctors’ lectures with the students and checked their notes.^45^

By the late 1870s, hygiene and cleanliness remained an important part of the taught instruction for nurses. Yet, consistent with Bashford’s findings at a general level, instruction in hygiene and cleanliness at St Thomas’ and the RIE was supplemented by a new emphasis on antiseptics and disinfectants. The limited amount of information about germ theories Croft and Bell disseminated to probationers at St Thomas’ and the RIE was not, however, an attempt to retain hospital hierarchies, nor to restrain intellectually upstart nurses, but resulted from their desire to raise nurses’ level of *practical* knowledge of infection control to a sufficient degree to allow nurses to conduct practices of wound dressing effectively in the wards and operating theatres. Both Croft and Bell deemed it necessary only to highlight to probationers the level of uncertainty surrounding the pathology of wound sepsis, rather than outline the details of those uncertainties. Both argued that ‘surgical cleanliness’ was the best preventative and curative measure for wound infection.

## Antisepsis, Asepsis and the Limits of Bacteriological Knowledge in Nurse Education at Glasgow Royal Infirmary and King’s College Hospital, London, 1880–1900

By the 1890s, an increasing acceptance of the existence and nature of micro-organisms among surgeons led to a growing recognition of the importance of scrupulous attention to the thorough disinfection of the patient’s skin, the hands of the surgeon and the nurse, instruments, sponges and ligatures, and dressings. Accordingly, definitions of ‘anti-sepsis’ expanded beyond Lister and his system symbolised in the 1870s as ‘spray and gauze’; surgeons increasingly adopted a combined antiseptic and aseptic approach to wounds and surgical cleanliness.^46^ Yet, while a greater consensus about micro-organisms as the causative agents of wound sepsis and the practices of wound management emerged, this consensus was not reflected in taught nurse education at the four hospitals. With nurse training still in flux, new antiseptic and aseptic content introduced into the lecture syllabi continued to vary according to the preferences and views about nursing education of the medical instructors.

Fierce debates over the establishment of mandatory classroom-based courses for probationers before they set foot in the wards resulted in the establishment of the first such course at the GRI. Supported by the GRI surgeon William Macewen and influenced by her training at the Nightingale School, the Matron and former Nightingale, Rebecca Strong, set up the first Preliminary Training School (PTS) for fee-paying nursing students at the nearby St Mungo’s College in 1893. Macewen, taught by Lister and a pioneer of asepsis, had been boiling instruments since the 1870s when the nurses on his ward purportedly clubbed together to buy him a fish kettle for the purpose, after the Infirmary managers refused. He relied on nurses in both the operating room, and the ward, where their careful continuous observation of his brain surgery cases was especially important. In a lecture *Nurses and Nursing* at the GRI in 1891, Macewen speaks eloquently of his indebtedness to nurses and a revolution in nursing, advocating raising nursing ‘to a distinct profession, with its entrance examination, its minimum requirements, theoretical and practical, its teachers, its examiners and its diploma’. He concludes that:

a nurse ought to be able to carry out an order with absolute fidelity, not in the mere letter, but in the spirit of the instruction. … A nurse ought to know the broad features at least of the disease she is fighting against. … Under these circumstances, instead of a mere automatic machine, the surgeon leaves a part of his brain beside the patient, and his treatment will therefore be faithfully carried out.^47^

The PTS arose from Macewen’s and Strong’s firm view that more classroom-based teaching would provide nurses with a ‘system of theoretical instruction’ necessary for practice.^48^ The College had already established courses and classroom facilities for educating medical students and allowed PTS students to take classes of anatomy, physiology, hygiene, surgical nursing, medical nursing and ward work, with demonstrations. It was also no coincidence that Strong was among the first nurses to publish a nursing textbook, a growing genre of textbooks written by the first generation of nurses who completed their training at the new training schools and who sought to pass on their knowledge of increasingly complex wound care systems to subsequent generations of nurses through taught instruction.^49^

**Figure 1.**
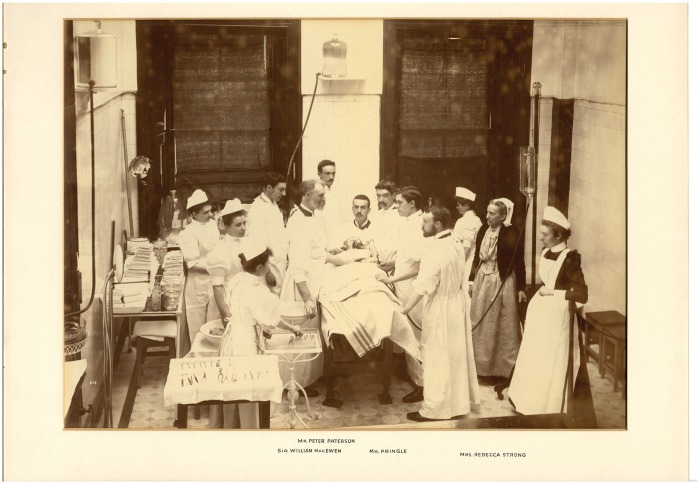
An operation at GRI conducted by William Macewen, which highlights the importance of assistance provided by both medical men and nurses, including Matron Rebecca Strong (second from right), c. 1890. With permission of NHS Greater Glasgow & Clyde

James A. Adams, surgeon at GRI and nursing instructor, developed and delivered the surgical nursing course of the PTS with assistance from Strong. He adopted a combined antiseptic and aseptic approach, similar in content to the courses delivered by surgeons to the hospital’s medical students.^50^ According to his 1895 syllabus, Adams dedicated over half of his eight-part course to daily practical lectures and demonstrations about types of wounds and their dressings, ulcers and their treatment, antiseptics, and the nature, causes and treatment of wound sepsis; he used practical and written examinations to test students’ understanding of wounds and their healing.^51^ The corresponding lecture notes of PTS probationers, Margaret Williamson in 1895 and Daisy Cosby in 1899, highlight Adams’ recommendation for nurses to vary their use of chemical disinfectants or steam for sterilisation according to the particular circumstances and subjects involved.^52^ His discussion of different types of antiseptics, their properties and use is extensive—he recommends carbolic acid as ‘the best all round antiseptic’—and combines this with advice that aseptic practice should be followed where possible through the regular changing of dressings and cleaning the operation room ‘because then there is no need for antiseptics’.^53^ This content was reinforced in ward work, where nurses were taught the practicalities of applying different kinds of dressing. While many surgeons, including Joseph Bell at RIE, began to discourage nurses’ use of sponges in the wards and in operations due to their potential for harbouring germs, Adams taught nurses how to clean and sterilise them.^54^

Crucially, Adams’ discussion of aseptic practice, particularly sterilisation, featured alongside the first definitive discussions of microbes as disease causing agents within nurse training at these four hospitals, and is one of the first appearances of ‘surgical bacteriology’ aimed at nurses. Adams describes different types of bacteria—streptococcus and staphylococcus—as very dangerous and infectious, and introduces probationers to the process that destroys them by heat ‘called sterilisation. … The apparatus for the purpose is a steriliser’.^55^ Hanley has similarly dated the first appearance of bacteriological causation in instruction for midwives about *Ophthalmia Neonatorum* in 1895. Micro-organisms appear only slightly later in the nursing and midwifery curricula than in the medical curricula. Indeed, it was only in 1887 that the surgical laboratory at Edinburgh University explicitly offered teaching in bacteriology for medical students.^56^

Yet, Adams’ surgical bacteriology for nursing students had several crucial differences to bacteriology aimed at medical students. First, Adams began to use accessible metaphors to convey bacteriological concepts to nurses. For example, by 1899, Adams was delivering a separate introductory lecture about microbes as lecture five and referred to their differing ‘life histories’. Matron Strong also supplemented these lectures by giving classes that briefly outlined how and why septic wounds contained microbes. Second, Adams’ combined antiseptic/aseptic approach, reinforced, rather than replaced, familiar regimes of hygiene. Reflecting a continuity of instruction over the previous two decades, the emphasis remained on the practical aspects of how to destroy bacteria with antiseptics and on ‘perfect, absolute cleanliness and attention to the most minute points’. What was new, however, was the incorporation of detailed sterilisation procedures into this system of disinfection and cleanliness. Finally, Adams emphasised personal cleanliness in his first lecture. He reiterated the importance of nurses having clean hands several times. Cosby’s lecture notes for his first lecture boldly state: ‘*Dirty hands—very important*’; lecture twelve contains the admonition: ‘Take great care to have hands thoroughly cleansed, do nothing, or touch nothing else, if you have to touch anything go and cleanse your hands’. Like other hospitals in this period, the GRI urged trainee nurses to perfect their own standard of hygiene, particularly by scrubbing their hands, before they began to practise aseptic cleanliness on the wards and in the theatre. This formed part of a broader precondition of caring for others and features prominently in the advice literature in nursing.^57^ While it is unclear to what extent such standards of personal cleanliness were impressed on medical students at Glasgow, elements of these standards may have been included in the Institutes of Medicine lectures on ‘Hygienical Sciences’ or in Professor Gairdner’s lectures on acute specific fevers ‘in treating of which the general doctrines of contagion and infection will be discussed’.^58^ However, the fact that personal hygiene had long been intimately bound up with the moral character of the nurses suggests they were more heavily impressed in lectures to nurses.

Meanwhile, the continued preference for antiseptic systems over asepsis at KCH during the 1890s, presumably due both to Lister’s position as Professor of Clinical Surgery until 1892 (succeeded by one of Lister’s most loyal disciples and author of *Antiseptic Surgery*, William Watson Cheyne) and to the lack of a PTS until the 1920s, resulted in a different type of taught instruction for nurses compared to the GRI.^59^ KCH integrated hour-long lectures for paying nurse probationers into a number of courses in 1882, but it was the surgical nursing course beginning in the 1890s and run by the Hospital’s well-respected surgeon and medical and nursing instructor, Albert Carless (1863–1936), that provides the best evidence for KCH’s continuing preference for antiseptic systems. Existing course syllabi for consecutive years between 1892 and 1898, reveal that Carless taught nurses how to treat instruments in antiseptics in at least four out of eight of his lectures. George Lenthal Cheatle (1865–1951), an assistant surgeon to the Hospital who taught courses alongside Carless dedicated two of his twelve surgical lectures to antiseptics; Cheatle went on to develop his eponymously named forceps for removing sterile items from sterilisers and changing dressings and in 1896, called for a closer relationship between bacteriology and clinical surgery.^60^

**Figure 2.**
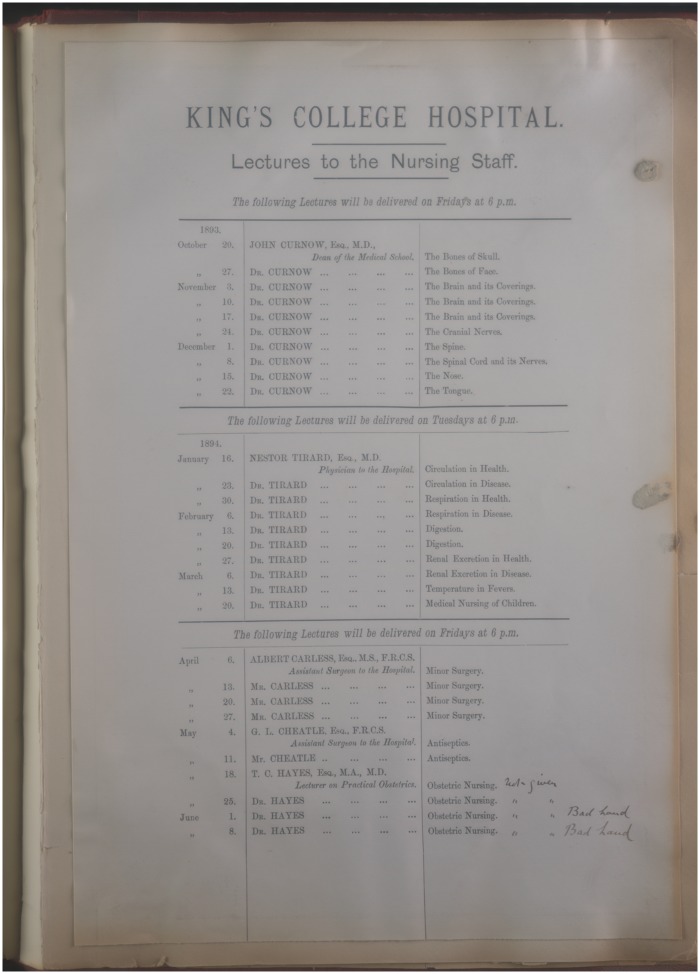
King’s College Hospital’s surgical nursing syllabus of 1893 included several lectures by Arthur Lenthal Cheatles on antiseptics. With permission of King’s College London Archives

Carless’ support for antisepsis and its originator Lister was well known in medical circles. Not only did he serve as an assistant to Lister in 1889, and as a colleague of Watson Cheyne, but his preference for antisepsis also appears in his *Manual of Surgery*, a textbook he co-authored with fellow KCH surgeon William Rose and first published in 1898. The *Manual* was aimed at KCH medical students, but its emphasis on antisepsis, and the practical nature of its system, may also explain why Carless recommended it to his probationer nurses too.^61^ The authors, like several other high profile surgeons of the period, viewed antisepsis as more reliable and easier to practise than asepsis, particularly in large city hospitals.^62^ In his report on cases of interest for the year in 1893/4, Carless stated that opening the knee under asepsis was still ‘a measure fraught with risk in the hands of those who cannot maintain the wounds they have made in an aseptic state’.^63^ Rose and Carless also nostalgically dedicated the 9th edition of their *Manual* to ‘Lord Lister, who first applied to surgery the principles that were being taught by Pasteur as to the microbic origin of disease’, and describe Lister as ‘one of the greatest benefactors of the human race’.^64^ In addition, the book contains approximately 20 separate references to Lister and 73 mentions of the word ‘carbolic’, the recommended antiseptic for handwashing, the washing out of wounds, instruments, sponges and sutures, and for cleansing the operating theatres, despite the greater success many other surgeons were having with other disinfectants.^65^

Another key text recommended to KCH nurses, John Kennedy Watson’s *A Handbook for Nurses* first published in 1899, also strongly favoured antiseptic approaches and the use of chemical disinfectants over preventive cleanliness. While emphasising personal cleanliness and discussing the care and complications of wounds across three out of eleven chapters, Watson maintained that asepsis could not be totally relied upon.^66^ Watson’s praise of Lister for developing his system of treatment following his recognition of the existence of germs in wounds appears excessive compared to other nursing textbooks published in the 1890s and may be a reason KCH chose to recommend his book. Watson drew the nurse reader’s attention to carbolic lotion in which to soak boiled sutures, perchloride of mercury and iodoform, as well as Lister’s later innovations and adaptations, such as the mercury and zinc compounds he recommended as an antiseptic in 1889, together with cyanide gauze for dressing wounds. The *Handbook* explained that the nurses’ hands should be thoroughly washed and scrubbed with a nail-brush, and then well rinsed in an antiseptic before she brings them into contact with a wound; the patient’s skin must be similarly prepared for an operation with an antiseptic.^67^ Watson’s dedication of his *Handbook* to ‘Professor Chiene, my esteemed teacher’, suggests Lister’s influence, as John Chiene, Professor of Surgery at Edinburgh University from 1882 to 1909, had been a public champion of mainstream Listerism since at least 1870.^68^

Watson’s emphasis on antisepsis also corresponds with his view of the need for doctors to maintain a dominance over nurses and over bacteriological knowledge in particular. The preface of his *Handbook* mentions the ‘vexed question about how much medical knowledge we should impart to nurses’, and states that experienced medical instructors like himself should disseminate to nurses only ‘a certain amount of medical knowledge’, while chapter 19 on the duties of a nurse in relation to operations and the care of wounds asserts that nurses should only be exposed to scientific knowledge in order to offer the surgeon ‘intelligent aid’.^69^ Limiting the discussion of bacteriology was Watson’s way of preventing what he feared would be ‘the illegitimate use of such knowledge’. While Watson’s views on the ways in which nurses could illegitimately use such knowledge remain unclear, such insights into the relationship between his preference for antisepsis and limits placed on the dissemination of bacteriological knowledge to nurses suggest a greater degree of tension at KCH between medicine and nursing than existed at St Thomas’ and RIE in the 1870s and at the PTS at the GRI in the 1890s. While antisepsis remained the focus of taught nursing instruction at KCH, the dissemination of bacteriological knowledge to nurses by the 1890s appears to have been more circumscribed.

## The Development of PTS, Practical Training and Bacteriology at St Thomas’ and KCH, 1900–1920

Between 1900 and the introduction of the first national standardised state examinations for nurses in 1923, a growing number of nurse training schools established PTSs, including St Thomas’ in 1910, and KCH and RIE in the early 1920s. The establishment of these schools allowed specially appointed sister-tutors to deliver more classroom-based training alongside medical and surgical lectures and this affected not only the balance between taught and practical nursing instruction, but also the ways in which knowledge about aseptic techniques and bacteriology was incorporated into curricula. Syllabi for taught lecture courses and corresponding examination papers used to measure the level of knowledge among nurses indicate a continuing emphasis on personal cleanliness and a wide acceptance of the combined antiseptic/aseptic approach to wound management; they also highlight a continuation of varied approaches to bacteriology at each of these hospitals.^70^ The changes reflected the growing importance of the ability of the well-educated nurse to support the surgeon by successfully conducting increasingly complicated observations and aseptic procedures, particularly as surgical ‘scrubber’ where the nurse’s tasks would include washing the operating theatre, boiling instruments, coiling and soaking a range of ligature and suture material.^71^

Adams’ surgical nursing course at the GRI in 1906, where the PTS had been established for almost ten years, continued to integrate discussions about the varieties of bacilli with their causative role in suppuration, abscesses and hospital diseases, together with methods of destroying these organisms.^72^ Yet, Adams never examined nurses on the science of bacteriology; instead, he used his annual one-and-half hour examination to probe nurses’ practical knowledge about the properties of different antiseptics and germicides, surgical dressings, preparation for operations, wounds and their healing, erysipelas and abscesses, and about sterilisation as a method of destroying organisms alongside antiseptics. Written examinations at St Thomas’ on surgical nursing and elementary science for special probationers from 1900 similarly avoided testing probationers on bacteriology, and instead, tested their practical knowledge of how to prepare the patient’s skin for an operation and the dangers of failing to do so; what they understood by the clinical terms ‘gangrene’ and ‘abscess’; the chief local and general signs pointing to wound infection; and the disinfecting qualities of both ‘superheated steam’ and ‘saturated steam’.^73^ Answers on the causative nature of germs in wound sepsis were not required. Marion A. Gullan, appointed in 1914 as the first sister-tutor for the newly established PTS at St Thomas’, with a role distinct from the home sister, developed more practical classroom-based sessions for PTS probationers; as a result, medical and surgical staff lectures took place after probationers left PTS, with supervision by Gullan. Notes from these lectures by Miss Coode, a probationer, suggest that by 1919 instruction in bacteriology formed part of a lecture course on biology in the second year of training, rather than in Gullan’s PTS. Coode’s biology lecture notes explain that the purpose of bacteriology was to treat the influence of micro-organisms on putrefactive and pathogenic processes before going on to outline the varieties of bacteria and instructions on how to prepare a microscope slide, all without once mentioning the wards or the practical responsibilities of the nurse.^74^ It is, however, unlikely that Gullan neglected all mention of bacteria in the PTS. While notes from her PTS are wanting, her *Theory and Practice of Nursing* first published in 1920, underpinned much of nursing instruction at St Thomas’ into the 1960s, and highlights her view that nurses should know how micro-organisms affect practice. In the first chapter, Gullan drew on the presence of micro-organisms in wounds to emphasise the nurses’ need to destroy them using antisepsis and asepsis, including dry and wet sterilisation.^75^

Like Croft in the 1870s, Gullan’s omission of detail on microbes, their structure and taxonomy may have resulted from a view that this knowledge was unnecessary for effective aseptic practice among nurses. Yet, she might also have recognised that Nightingale probationers had not been responding well to decades of taught instruction from physicians and surgeons. Like Crossland in the 1870s, Gullan emphasised both what she felt probationers needed to know, and how they could come to know it. Within the *Theory and Practice of Nursing*, Gullan advised probationers to consult the publication regularly for revision throughout their training, and included six blank leaf pages for every 15 pages of text in order for probationers to make their own revision notes, as well as making notes or illustrations of their own clinical experience. Moreover, the positive reception of Gullan’s changes to the structure of nurse training among Nightingale probationers suggests that they had been dissatisfied with previous medical instruction. Probationer Boycott described Gullan’s classes as a valuable training development because it allowed ordinary probationers to obtain crucial practical knowledge before they entered the ward.^76^ Probationer Ovans viewed formal instruction from medical men as far less important than practical instruction; she described lectures and examinations as ‘rather a farce’.^77^ Other probationers, particularly the ‘ordinaries’, reported that they had barely paid attention in their hour or two a week of formal lectures, some had fallen asleep and others felt it unfair that these lectures were scheduled during their time off from ward work.^78^ By the early 1900s then, the demarcation between medicine and nursing at St Thomas’ was not only maintained by some medical men, Nightingale, and other opponents of nursing reform, but also by the School’s probationers themselves.

In contrast, the RIE’s decision not to establish a PTS in this period following lengthy study of nursing provision at other hospitals in 1902 meant that its training continued to consist of an unsystematic programme of practical instruction by Lady Superintendent Annie Gill and her assistants with only sporadic formal lectures by hospital staff like Bell.^79^ The sporadic training programme meant that the content of instruction on wound sepsis was largely integrated into scheduled ward work.^80^ Indeed, while lectures did emphasise that pathogenic explanations of wound sepsis ‘was of the most importance to nurses’, Gill argued in 1907 that lectures could only ever provide a summary of a topic, while the real understanding came from ward work.^81^ Similarly, lectures given by Alexander Hugh Freeland Barbour (1856–1907), RIE gynaecologist, revealed his desire to maintain professional hierarchies by asserting that nursing was a vocation and the discipline of home life was the best form of training a nurse could receive: ‘Remember that the foundation-qualities of a good nurse, gentleness, patience, and reverence come from home discipline. Gentleness, patience, reverence: these are the original elements in the make-up of a good nurse. They are the roots of character. And they are yours by inheritance’.^82^ Its decision not to establish a PTS suggests that Bell’s preference for lecture based learning in the 1870s was not shared by many at the RIE.^83^

At KCH, Carless’ relatively late incorporation of a combined antiseptic/aseptic approach within his surgical nursing course is reflected in his syllabi and examination papers between 1902 and 1918, which coincided with the establishment of a more PTS-based model training school at the hospital in 1906.^84^ While a PTS at KCH was not officially established until the 1920s, the hospital did employ a home sister, Wolseley-Lewis, to provide junior probationers with instruction in asepsis and antisepsis to supplement Carless’ lectures on the topic. At least two out of the five questions on each of the hour-and-a-half examination papers set by Wolseley-Lewis and by Carless were a direct test of nurses on lecture topics—‘sepsis, antisepsis and asepsis’, ‘wounds and their repair’ and ‘suppuration’. The questions assessed their clinical knowledge of the healing of wounds and the development of abscesses, and their practical preparations for an operation and treatments for septic wounds. Indicative of his acceptance of the increasing importance of asepsis in nursing practice at KCH, Carless tested probationers on sterilisation for the first time in 1909, by asking them to explain how the nurse should prepare dressings, towels and swabs for an aseptic operation if (a) a steriliser is available and (b) if a steriliser is not available. Carless’ late incorporation of sterilisation into his syllabus at KCH is striking because nurses at St Thomas were using an autoclave to sterilise dressings by 1894 and Adams introduced sterilisation to probationers at GRI in 1895.^85^

Similarly, the style of Carless’ examination questions changed, indicating a move in nursing education further away from passive classroom-based learning, towards active practice-based learning. As Russell Howard at the London Hospital asserted in his 1908 surgical nursing textbook, active strides in surgery required greater knowledge on the part of the nurse.^86^ Accordingly, an increasing number of Carless’ examination questions became scenario-based, in order to better test a nurse’s initiative, skills and attention to detail as would be needed in the wards, operating theatre and beyond.^87^ Carless’ examination paper for 1908, for example, asks:

You arrive at a Patient’s house in the country, and are told to prepare as quickly as possible for an emergency abdominal operation (say for perforated gastric ulcer). A small chemist’s shop is in the village, but the only dressings available are unsterilized gauze and absorbent wool. The surgeon is expected in two or three hours. How would you set to work to get things ready?

To accompany the problem-based learning approach as a way of testing nursing knowledge, textbooks began to incorporate tests at the end of each chapter, some of which hospital examinations replicated word for word. A question on the differences between asepsis and antisepsis, for example, was among those on Carless’ KCH examination of 1909 and among seven test questions including in Watson’s *Handbook* of 1912 after chapter eleven on inflammation.^88^

**Figure 3.**
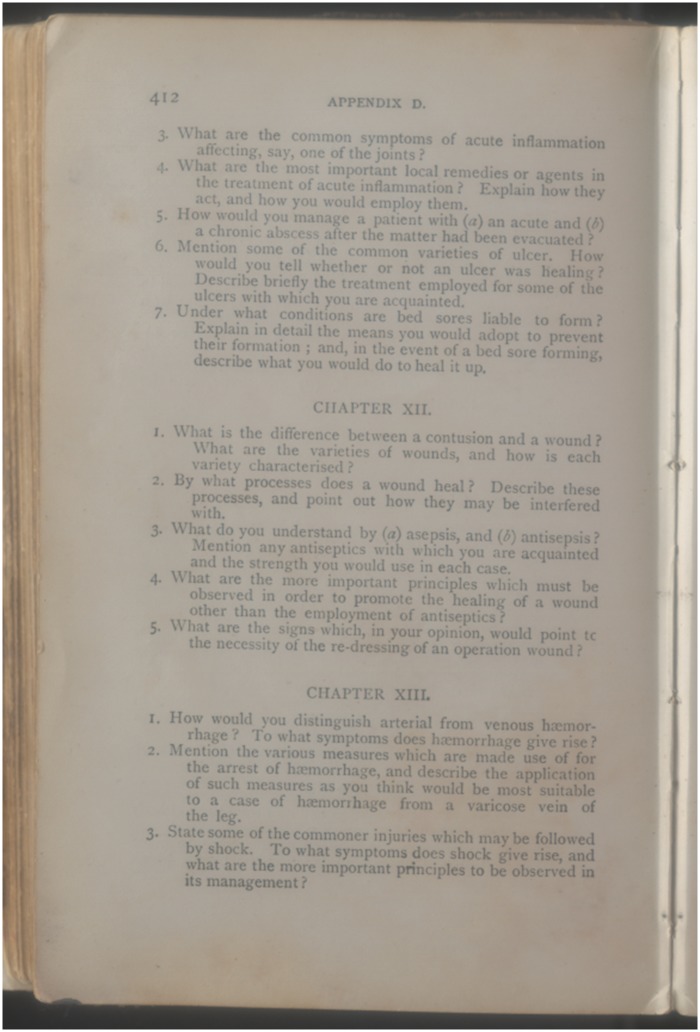
Watson’s Handbook for Nurses of 1912 included example examination questions, including those for wound sepsis. With permission of King’s College London Archives

Carless’ adoption of an antiseptic/aseptic approach also led him to attempt to re-address not only the amount of bacteriology he included in nurse training, but also when it would be introduced. Carless’ new emphasis on the importance of ‘careful microscopic and bacteriological investigation of wounds’ and on the closer relationship between clinical and pathological aspects of a case appear in his early twentieth-century reminiscences.^89^ In 1903 he attempted to introduce surgical bacteriology before clinical symptoms in his surgical nursing course by beginning the course with a guest lecture on ‘the germ theory of disease’ by Norman Dalton, one of the hospital’s physicians, but he abandoned the guest lectures in 1906. Carless waited until 1913 to reintroduce lectures on ‘bacteriology’ into the nursing syllabus—J. C. Briscoe, assistant physician to the hospital delivered two lectures on bacteriology, while Dr Gilliatt and W. D’Este Emery, the hospital’s lecturer in pathology and bacteriology, delivered three – but these lectures appeared at the end of the course, rather than the beginning as in 1903, in order to reinforce practical points. The lack of change in the syllabus for at least a decade thereafter indicates the success of his reintroduction of a structure that began with practical instruction followed by bacteriological content.^90^ Like Adams at the GRI, Carless set no questions on germs in any of his examination papers. He may have felt that probationers, like those at St Thomas’, struggled to answer bacteriological questions and their knowledge of this topic would be tested in other ways. However, the inclusion of lectures emphasising the pathological aspects of wound sepsis by Dalton and D’Este Emery on his course nonetheless suggests that Carless felt that it was important for those with the highest level of bacteriological expertise to disseminate what they knew to nurses.

While Carless has left little explicit record of his motivations for curriculum changes, comparison of his syllabi for his surgical nursing course and for his surgery course aimed at KCH’s medical students, along with amendments made to the 1914 edition of his co-authored *Manual*, highlight both his commitment to further incorporating surgical bacteriology into nursing, and his view that the best way to do this was to shape nursing education more in line with medical education. Both lecture one of his surgery course for medical students and chapter one of the *Manual*, written by D’Este Emery, addressed surgical bacteriology, infection and immunity, while inflammation formed the content of lecture and chapter two. The principles and practices of asepsis and cleanliness adapted to suit current practice (with warnings about the irritating properties of carbolic, the introduction of cotton gloves and overalls, and consideration of the layout and design of the operating room) were embedded into these two lectures/chapters.^91^ While this structure was at odds with his new nursing syllabi, Carless continued to recommend the *Manual* to nurse probationers. Carless’ personality and reputation among nurses in this period as strict and impatient may have driven him on to make these changes regardless of the reception they received among probationers.^92^ In contrast, Watson’s 1912 edition of the *Handbook* maintained its practical emphasis over pathology in order to shape nurses’ medical knowledge. Watson’s incorporation of further details on asepsis, for example, and on ways to clean the operating theatre, expanded to almost 50 out of 500 pages, and demonstrated his renewed commitment to aseptic principles, and contained less information about sepsis causation.

The differences between the taught curricula of different hospitals and between medical students and nursing probationers notwithstanding, it is clear that by the 1919 Nursing Registration Act nurses had become more active in their own learning and like medical students, withdrew books from their hospital’s library, purchased their own textbooks, and demonstrated their ownership of their books by writing their name in pen down the side of the pages or inside the front cover.^93^ ‘Miss J. J. Murray,’ for example, signed the inside front cover of the 1919 edition of A. Millicent Ashdown’s *A Complete System of Nursing* in the KCH Library. This text, written by a sister tutor in bandaging and practical work at KCH, underpinned much of the nursing instruction into the twentieth century and went into multiple editions into the 1960s. Her expression of sincere thanks to Carless, among other hospital medical staff, for rendering assistance and giving advice also suggests the continued reliance of nurse education on medical instructors.^94^

**Figure 4.**
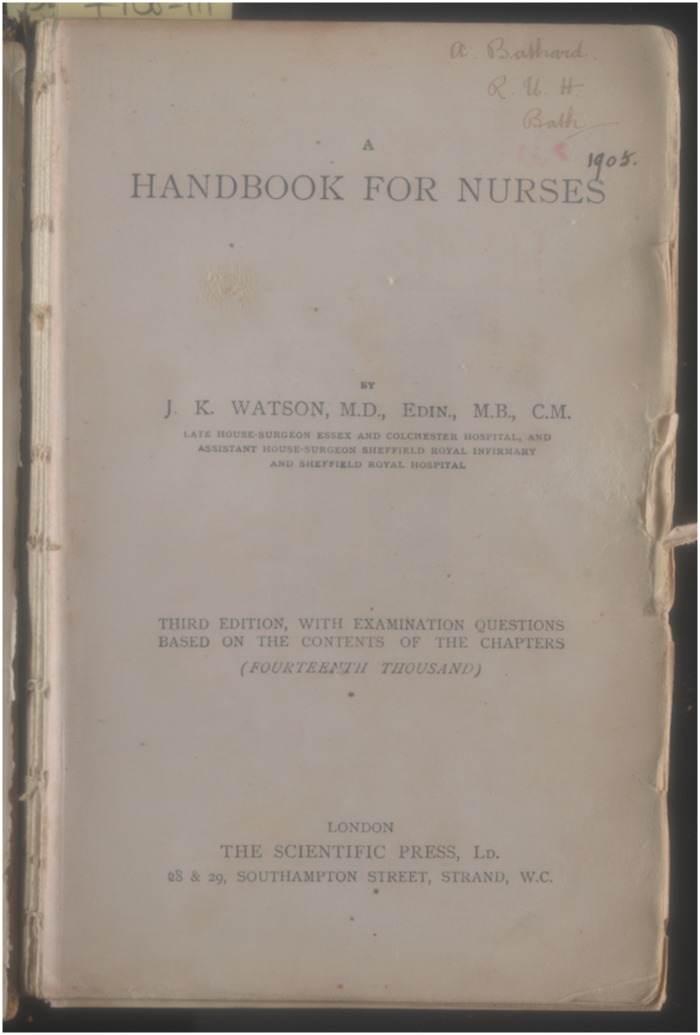
Watson’s Handbook for Nurses of 1905 is signed by an ‘A Bathard’ of Bath. With permission of King’s College London Archives

By 1920, medical instructors achieved more success when they demarcated their instruction for medical and nursing students by shifting the emphasis on bacteriology between practical and theoretical sections. Yet, debates over the appropriate balance of clinical and pathological knowledge within surgery and wound management were far from over. The introduction of the nursing register in 1919 did little to standardise the curricula; hospitals continued to set their own syllabi and examinations, alongside those that were set by the national governing body from 1924.^95^ Meeting minutes from 1923 at St Thomas’, for example, suggest that medical and surgical instructors were concerned about the low standard of knowledge resulting from inadequate training, revealed in nurses’ examinations. Some sisters expressed concern that theoretical nursing was encouraged at the expense of practical teaching and that nurses spent so much time over study that they came to the wards tired and unreceptive, while Miss Coode argued that some probationers were unhappy about their lack of knowledge on commencing ward work.^96^ Debates over the balance between practical and taught instruction prevailed into the era of sulphonamides and antibiotics in the 1930s and 1940s, as surgeons increasingly viewed nurses as members of their surgical team.^97^

## Conclusion

By examining the provision of nursing education at four of Britain’s leading teaching hospitals between 1870 and 1920, it is clear that the principles and practices used for preventing and managing wound sepsis not only constituted an important part of ward work and practical training, but also formed an integral part of taught instruction by hospital medical and surgical staff and associated tutors. As preventing and managing wound sepsis became an increasingly important responsibility of the new type of nurse at the newly established training schools, the increasing incorporation of instruction about disinfectants in the 1870s, surgical cleanliness in the 1880s and 1890s and sterilisation from 1900 is no surprise. Yet, prior to the establishment of national standards of training, each of the hospitals and their medical instructors had the freedom to dictate how much of this instruction to include, when to introduce it, how to teach it and the balance to strike between practical and theoretical instruction and between clinical and pathological knowledge. This freedom resulted in varied provision across the four hospitals, and even the same instructor could change his opinions over time. In the 1870s, Croft at St Thomas’ and Bell at RIE maintained their emphasis on hygiene while incorporating new disinfecting principles and practices, and were willing to share the theoretical ambiguities with nurses surrounding germ theories. By the late 1890s, Adams at the newly established PTS at the GRI instructed nurses in a combined antiseptic/aseptic approach, which incorporated aspects of the new surgical bacteriology and how it underpinned nursing practices; while Carless’ preference for antiseptics at KCH resulted in a continuing emphasis on the practicalities of destroying bacteria. With the consolidation of asepsis and bacteriology in the 1900s and the more widespread establishment of PTSs, Carless changed his emphasis to combine antiseptic and aseptic approaches, so that the underpinning germ theories of infection appeared alongside practical methods for disinfection and sterilisation. At the same time, he attempted to shape instruction for surgical nursing along similar lines to his surgery course for medical students.

At the centre of these variations in content were questions about the nature of nursing and what form it should take following the establishment of the new style of nurse training school. Fluctuations and tensions surrounding the appropriate balance between formal taught lectures, accompanying examinations and textbook learning, along the lines provided for medical students, and practical ward work and instruction, represented concerns over whether nursing should be seen as a profession along similar lines to medicine, or as a vocation. What we demonstrate is that the content of knowledge and practice were much more a process of negotiation between doctors and nurses operating in localised systems of health care and those they networked with more broadly, certainly in the case of wound sepsis. This was especially the case during the early part of the period when bacteriological aetiology was still being debated within the scientific community. Furthermore, there were differences in the degree to which asepsis and antisepsis were assigned priority in debates. Underpinning all was the assumption and assertion that the hygienic nurse, her practice in terms of her own personal hygiene and scrupulous attention to the hygiene and cleanliness of the ward and wounds were a fundamental underpinning for asepsis and antisepsis to be successful, thereby securing the safety of hospitals and reputations of the medical profession who practised within them and trained increasing numbers of medical students. It remains to be seen whether similar processes of negotiation between doctors and nurses over knowledge and practice took place in other areas of medicine, particularly those in which nurses’ interventions were less crucial.

In lieu of further research on doctor–nurse negotiations, we see the boundaries between medical and nursing practice as fluid and shifting shape in different organisational environments at different points in time. The practical and apprentice-like training aspect of nursing and its embodiment of moral character, typically discussed by nursing historians, was undoubtedly important, and represented a contrast to medical instruction. Yet, instruction about the principles and practices surrounding wound sepsis, the increasing production of textbooks specifically aimed at nursing probationers by both medical and nursing instructors, the increasing number of examinations, the move away from a prescriptive style of examination to a more scenario-based test, more formalised syllabi, and the establishment of PTSs, were symbols of support for a more classroom-based teaching model of nursing along the lines of medical education, and represented a flexibility in the balance between theory and practice. The pedagogical tools did not necessarily represent a desire for medicine to maintain its gendered professional hierarchy, although clearly for those like Watson it did. Instead, they demonstrate how much taught instruction depended on the practitioners’ changing level of knowledge, their preferences, the practical nature of topics, such as disinfection and cleanliness, and simply, the amount of time set aside for taught instruction. Of course, the similarities between the content and form of instruction at all four hospitals suggest a growing consensus about medical knowledge around infection and its control, but the contrasts indicate that there was little ‘scientific truth’ to disseminate, rather the preferences of knowing individuals.

## Footnotes

1 John S. Bristowe, ‘How far should our hospitals be training schools for nurses?’ [1878] HO1/ST/NTS/Y/87/001, 9, 13, St Thomas’ Hospital Archive, London Metropolitan Archive (henceforth StTH).

2 Gender historians have typically focused on the Victorian concept of ‘separate spheres’. For implicit endorsement of the ‘separate spheres’ thesis in nursing, see, for example, Brian Abel-Smith, *A History of the Nursing Profession* (London: Heinemann, 1960 reprinted by Ashgate, 1992); Lucy Seymer*, Florence Nightingale’s Nurses: The Nightingale Training School, 1860–1960* (London: Pitman Medical Publishing Company, 1960); Roy Wake, *The Nightingale Training School 1860–1996* (London: Haggerston Press, 1998).

3 Alison Bashford, *Purity and Pollution: Gender, Embodiment and Victorian Medicine*, (Basingstoke: Palgrave Macmillan, 1998), xiii. Others have acknowledged more fluid boundaries between nursing and medicine but have yet to explore the topic in any depth. See, for example, Monica E. Baly, *Florence Nightingale and the Nursing Legacy*, 2nd edn (London: Wiley, 1998), 35, 156; Julia Hallam, *Nursing the Image: Media, Culture and Professional Identity* (London: Taylor & Francis, 2002); Carol Helmstadter, ‘Authority and Leadership: The Evolution of Nursing Management in 19th century Teaching Hospitals’, *Journal of Nursing Management*, 2008, 16, 4–13; Anne Marie Rafferty, *The Politics of Nursing Knowledge* (London: Routledge, 1996); Michael Worboys, *Spreading Germs* (Cambridge: Cambridge University Press 2000), 12.

4 ‘Four sheets of manuscript notes by Nightingale on the report of the committee appointed to inquire into the sanitary state of St Thomas’, [1878] A/NFC84/002, 2, StTA. Bristowe seemingly uses ‘principles’ to refer to his assumptions about pathology, assumptions he shared with other physicians and surgeons of this period. Christopher Lawrence and Richard Dixey, ‘Practising on Principle: Joseph Lister and the Germ Theory of Disease’ in Lawrence, ed., *Medical Theory, Surgical Practice: Studies in the History of Surgery* (London: Routledge, 1992), 153–215.

5 Graham A. J. Ayliffe and Mary P. English, *Hospital Infection: From Miasmas to MRSA* (Cambridge: Cambridge University Press, 2003), esp. 68–117.

6 Rafferty, *Politics of Nursing Knowledge*, 24.

7 The Nightingale School at St Thomas’ Hospital was the first established in 1860, followed by schools at the twelve London teaching hospitals. Carol Helmstadter and Judith Godden, *Nursing Before Nightingale, 1815–1899* (Surrey: Ashgate, 2011).

8 Bashford, *Purity and Pollution*; Pamela J. Wood, ‘Supporting or Sabotaging the Surgeon’s Efforts: Portrayals of the Surgical Nurse’s Role in Preventing Wound Sepsis, 1895–1935’, *Journal of Clinical Nursing*, 2009, 18, 2739–46. For separate discussions of wound sepsis and nursing, see, for example Ayliffe and English, *Hospital Infection*; Abel-Smith, *History of the Nursing Profession*; Celia Davies, ‘A Constant Casualty: Nurse Education in Britain and the USA to 1930’, in Celia Davies, ed., *Rewriting Nursing History* (London: Croom Helm, 1980), 102–22.

9 Anne Hanley, ‘“Scientific Truth into Homely Language”: The Training and Practice of Midwives in *Ophthalmia Neonatorum*, 1895–1914’, *Social History of Medicine*, 2014, 27, 199–220. See also Hanley, *Medicine, Knowledge and Venereal Diseases in England, 1886–1916* (London: Palgrave Macmillan, 2016), esp. ch. 5.

10 Baly, *Florence Nightingale*; Lynn MacDonald, ed., *Florence Nightingale: The Nightingale School* (Waterloo, Ontario: Wilfred Laurier University Press, 2009); Rafferty, *The Politics of Nursing Knowledge*; Wake, *The Nightingale Training School.*

11 A striking example is the pioneering aseptic surgeon William [later Sir] William Macewen, in his lecture *Nurses and Nursing* at the Glasgow Royal Infirmary in 1891.

12 Rafferty, *Politics.*

13 Michael Worboys, ‘Practice and the Science of Medicine in the Nineteenth Century’, *Isis*, 2011, 102, 109–15; Thomas Schlich, ‘“The Days of Brilliancy are Past”: Skill, Styles and the Changing Rules of Surgical Performance, c. 1820–1920’, *Medical History*, 2015, 59, 379–403.

14 Worboys, *Spreading Germs*; Lindsay Granshaw, ‘“Upon this Principle I have based a Practice”: The Development and Reception of Antisepsis in Britain, 1867–90’, in John V. Pickstone, ed., *Medical Innovations in Historical Perspective* (London: Macmillan, 1992); Jerry L. Gaw, *A Time to Heal: The Diffusion of Listerism in Victorian Britain* (Philadelphia: American Philosophical Society, 1999); M. Anne Crowther and Marguerite W. Dupree, *Medical Lives in the Age of Surgical Revolution* (Cambridge: Cambridge University Press, 2007). For a broader analysis of the variations within the medical profession towards bodies of knowledge and practice, see Christopher Lawrence, ‘Incommunicable Knowledge: Science, Technology and the Clinical Art in Britain 1850–1914’, *Journal of Contemporary History*, 1985, 20, 503–20;

15 Crowther and Dupree, *Medical Lives*.

16 From 1860, Nightingale put into practice her ideas about the importance of hygiene, ventilation and meticulous record keeping at the Training School at St Thomas’ Hospital that bore her name, while the system implemented at King’s Training School for Nurses by Sister Matron Katherine Monk from 1885 was widely admired and copied by other hospitals.

17 Andrew Warwick, *Masters of Theory: Cambridge and the Rise of Mathematical Physics* (Chicago: University of Chicago Press, 2003).

18 For an example of how methods of communication were vital in shaping medical ideas, see Claire L. Jones, *The Medical Trade Catalogue in Britain, 1870–1914* (London: Pickering & Chatto, 2013).

19 Rebecca Strong, *Reminiscences* (Edinburgh: Douglas & Foulis, 1935), 5.

20 ‘Special’ or lady probationers were generally better educated than ‘ordinary’ probationers and paid a fee to attend. The non-paying nursing probationers (or ‘ordinary’ probationers) received most of their instruction via practical ward work, supplemented with occasional classes conducted by the home sister.

21 Bristowe, ‘How Far Should Our Hospitals Be Training Schools for Nurses?’; *RIE Annual Report*, 1878/9, 5.

22 ‘Syllabus of Mr Croft’s Lectures for 1873’, HO1/ST/NTS/C/13/1, StTH. Antiseptic methods were being used at St Thomas’ by 1871, after Sydney Jones excised a knee joint under carbolic spray and dressed the wound with carbolic gauze. T. H. Pennington, ‘Listerism, its Decline and its Persistence: The Introduction of Aseptic Surgical Techniques in Three British Teaching Hospitals, 1890–1899’, *Medical History*, 1995, 39, 35–60, 47.

23 ‘Mr Croft’s notes of lectures: Lectures xix–xxi 1873 and 1874’, H01/ST/NTS/C/35/003, StTA, 68.

24 Nightingale made extensive notes on these outbreaks, complete with a table outlining numbers of cases in different wards: ‘Manuscript notes by Nightingale “remarks on spread of disease”’, A/FNC84/2; ‘Rough table of erysipelas cases arising Oct 1878-July 1879’, A/FNC/083/014, StTH.

25 Joseph Bell, *Notes on Surgery for Nurses* (Edinburgh: Oliver and Boyd/London: Simpkin, Marshall and Co., 1887).

26 ‘Mr Croft’s notes of lectures’, H01/ST/NTS/C/35/003.

27 ‘Mr Croft’s notes of lectures: Lecture XIX *Disinfectants and Antiseptics*’, H01/ST/NTS/C/35/003, StTH.

28 ‘John Croft’, *Dictionary of National Biography*. As Lawrence and Dixey have pointed out, Lister’s own ideas about the pathology of wound sepsis changed between 1867 and 1883, moving from a germ theory of putrefaction to one of infection. Lawrence and Dixey, ‘Practising on Principle’. John Croft, ‘Two Cases Illustrating the Pathology and Operative Treatment of Simple Fracture of the Patella’, *The Lancet*, 25 July 1891.

29 Croft’s notes of lectures: Lecture XIX *Disinfectants and Antiseptics.* Emphases in original. Croft referred nurses to Tyndall’s lecture at the Royal Institution in 1871. John Tyndall, ‘Dust and disease’, *British Medical Journal*, 24 June 1871, 1:547, 661–2. For more on Tyndall, see Ruth Richardson, ‘Inflammation, Suppuration, Putrefaction, Fermentation: Joseph Lister’s Microbiology’, *Notes and Records of the Royal Society*, 2013, 67, 211–29, 214. For further information on disinfectants, Croft refers probationers to a Dr H Letheby ‘Lecture on the Right Use of Disinfectants’, *Medical Times and Gazette*, 1 November 1873.

30 See also MacDonald, *Florence Nightingale*, 16.

31 ‘Mr Croft’s notes of lectures: Lecture XIX *Disinfectants and Antiseptics* 11; Bell, *Notes on Surgery*, 37.

32 Bell, *Notes on Surgery*, 40, 54, 72, 74.

33 See, for example, Joseph Bell, *Cases Illustrative of the Antiseptic Use of Carbolic Acid, Read before the Medico-Chirurgical Society of Edinburgh, 7th April 1869* (Edinburgh: Oliver and Boyd, 1869), 1–9.

34 ‘James Syme’, *Dictionary of National Biography*.

35 Bell, *Notes on Surgery*, 53, 72.

36 Helmstadter, ‘Authority and Leadership’, 9; Seymer, *Nightingale’s Nurses*, 70. For the creation of the position of ‘home sister’ at the nurses home and the appointment of Mary Crossland and of Croft, see Mark Bostridge, *Florence Nightingale: The Woman and Her Legend* (London: Viking, 2008), 452–5.

37 ‘Mr Croft’s notes of lectures: Lecture XIX *Disinfectants and Antiseptics* 4, 5; Bell, *Notes on Surgery*, 53, 72.

38 Bristowe, ‘How Far Should Our Hospitals Be Training Schools for Nurses?; ‘Four sheets of manuscript notes by Nightingale’.

39 Bell, *Notes on Surgery for Nurses*, preface.

40 McDonald, *Florence Nightingale*, 894. Lucy R. Seymer, ‘Mary Crossland of the Nightingale Training School’, *The American Journal of Nursing*, May 1961, 61, 85–8; Seymer, *Nightingale’s Nurses*, 70–1. Croft’s commitment was also indicated by his 1876 book *The Theory and Practice of Medicine* and his public health work, as Medical Officer of Health for Camberwell.

41 Rafferty, *Politics*, 29; Hanley, ‘“Scientific Truth into Homely Language”’.

42 ‘Reading list prepared by Mr Croft for the probationers’, HO1/ST/NTS/C/13/2, StTH; Gaw, *A Time to Heal*, 61.

43 Granshaw,’Upon this Principle’, 37.

44 Seymer, *Nightingale’s Nurses*, 66.

45 McDonald, *Florence Nightingale*, 15.

46 Granshaw, ‘Upon this Principle’; Lawrence and Dixey, ‘Practising on Principle’; Worboys, *Spreading Germs*, 33, 168.

47 William Macewen, *Nurses and Nursing* (Glasgow, 1892), 7. See also, A. K. Bowman, *The Life and Teaching of Sir William Macewen: A Chapter in the History of Surgery* (London: William Hodge & Co., 1942).

48 Rebecca Strong, *Education in Nursing: An Address Given in London in 1895* (Edinburgh: Douglas & Foulis, 1927), 10.

49 Rebecca Strong, *Hints for Beginners in the Work of Nursing* (Glasgow, 1882). Strong’s publication was a pamphlet, but books in the 1890s, such as Isabel Adams Hampton’s (later Isabel Hampton Robb) *Nursing: Its Principles and Practice for Private and Hospital Use*, were often up to 500 pages. Isabel Adams Hampton, *Nursing: Its Principles and Practice for Private and Hospital Use* (Philadelphia: W. B. Saunders, 1893).

50 James A Adams (1857–1930), the fifth generation of a medical family, worked as demonstrator in anatomy at the University and surgeon in the outpatient dispensary of the GRI before his surgical skill led to his appointment as surgeon as successor to Macewen in 1892. He lectured in the extra-mural medical school on clinical surgery, on surgical diseases of children, as well as surgery for nurses.

51 ‘Nursing syllabus, lectures by, James A Adams GRI’, 1895, HB95/3/1/1, Greater Glasgow Health Board Archive, Glasgow University Archives and Business Centre (hereafter GGHBA).

52 References to ‘sterilisation’ and ‘sterilisers’ appeared in the British medical literature in the late 1870s and 1880s after John Tyndall’s experiments; early displays of commercial ‘bacteriological apparatus’ including sterilisers appeared at the British Medical Association’s annual meeting in 1886: Becker and Co of Maiden Lane supplied ‘a complete set … as used in Germany’, while Messrs Griffin and Son of Covent Garden showed ‘apparatus of English make’, *British Medical Journal*, 1886, i, 225–6. By 1891, Joseph Coats, lecturer in Pathology at Glasgow University was showing ‘an autoclave steriliser, for sterilising by steam under pressure’ at a meeting of the Glasgow Clinical and Pathological Society, *British Medical Journal*, 1891, ii, 1228.

53 Daisy Cosby, ‘Surgical Notes 1899’, 1899, HB95/10/1/18; Margaret Williamson, ‘Surgical lecture notes’, 1895, HB195/10/1/13, GGHBA.

54 Bell, *Notes on Surgery for Nurses*, 41. See also, John K. Watson, *A Handbook for Nurses* (London: The Scientific Press, 1899), 184. Wood, ‘Supporting or Sabotaging the Surgeon’s Efforts’.

55 Cosby, ‘Surgical Notes 1899’, 8 March 1899, Lecture five.

56
*Edinburgh University Calendar, 1887–88*, 305–6. Bacteriology permeated the medical and surgical curricula across the country at different times, but this is two years earlier than Charles Lockwood established a laboratory and taught what has been called the first course in practical bacteriology in Britain at St Bartholomew’s Hospital, London. For more on bacteriology at St Bartholomew’s Hospital, see Rosemary Wall, *Bacteria in Britain*, *1880–1939* (London: Pickering & Chatto, 2013).

57 Wood, ‘Supporting or Sabotaging the Surgeon’s Efforts’, 2741.

58
*Glasgow University Calendars 1871–2*, 57–8; *1881–2*, 70; *1887–8*, 73–4; and *1893–4*, 74–5 for Macewen’s lectures on surgery including a section on ‘infective processes and diseases dependent on bacteria and their products’. For Professor Christenson’s medical lectures on ‘Cleanliness’ in 1870 and on ‘demonstrations on the principles of antiseptic surgery’ in 1890 at RIE, see *Edinburgh University Calendars 1870–1*, 89–90, 93; *1891–2*, 340.

59 For more on Cheyne, see Worboys, *Spreading Germs*, 150–80.

60 George Lenthal Cheatle, ‘The Importance of Bacteriology in Clinical Surgery’, *Annual Report of the Medical and Surgical Departments at King’s College Hospital*, 1895/6, 37–42.

61 A 1916 edition was presented to KCH’s Nurses’ League in 1917 and remains part of its archive. Moreover, Rose and Carless suggested that nurses read their manual: ‘the usual routine followed at King’s College Hospital in undertaking an operation, or in the treatment of a wound’, William Rose and Albert Carless, *Manual of Surgery for Students and Practitioners* (London: Ballière, Tindall and Cox, 1898), 199.

62 Worboys, *Spreading Germs*, 190; Rose and Carless, *Manual of Surgery*, 201–2.

63
*Annual Report of the Medical and Surgical Departments at King’s College Hospital*, 1893/4, 225.

64 Rose and Carless, *Manual of Surgery*, 9th edn, 1914, 271.

65 See, for example, W. Bruce Clarke, ‘Treatment of Wounds with Izal’, *The Lancet*, 1 July 1893, 18–19.

66 Watson, *A Handbook*, 123. Like *A Manual of Surgery*, the 1905 and 1912 editions of *A Handbook for Nurses* was found in KCH’s Nurses League archive by which point it had become a key text.

67 Watson, *A Handbook* (1899; 1905; 1914), 115.

68 Worboys, *Spreading Germs*, 92.

69 Watson, *Handbook for Nurses* (1912), 179.

70 The first evidence that nurses sat written examinations as a way of measuring their knowledge appears at GRI in the PTS in 1895 and at KCH in 1896, although no separate surgical examinations seemingly exist until 1903.

71 Wood, ‘Supporting or Sabotaging the Surgeon’s Efforts’, 2742.

72 ‘Education in Nursing Syllabus’, 1906, 1908, HB95/3/1/3–4, GGHBA. See also the textbook recommended by Adams: Andrew N. McGregor, *A System of Surgical Nursing* (Glasgow: David Bryce & Son, 1905). McGregor was GRI’s assistant surgeon.

73 ‘Surgical Exam’, 2 July 1900, HO1/S/NTS/C/40/1, Nightingale Training School (NTS), London Metropolitan Archives; ‘Elementary science exam’, 1900, HO1/ST/NTS/C/40/10, NTS. At the Nightingale home from 1900, special probationers also sat written examinations in anatomy and physiology alongside other subjects including surgical nursing and elementary science.

74 ‘Lecture notebook belonging to Miss Coode 1918’, H01/ST/NTS/Y/54/001, NTS.

75 Marion A. Gullan, *Theory and Practice of Nursing* (London: H. K. Lewis, 1920), 1–10, 1.

76 ‘Recollections: C. Boycott’, 1900, H01/ST/NTS/Y22, NTS.

77 ‘Recollections: Lady Acland (née Ovans)’, 1895–1903, H01/ST/NTS/Y22, NTS.

78 For example, ‘Recollections: Corner (née Henderson)’, 1899, H01/ST/NTS/Y22, NTS.

79 It should not commit funds ‘to educate young women in preliminary subjects’. Report of the Special Committee on Preliminary Training of Nurses, etc’, 1902, LHB1/104/46, RIE, Lothian Health Board Archive Edinburgh (henceforth RIE).

80 Jessie Alexandria Cassels, ‘Notes of nursing lectures by Gill and Bladon’, October 1912, GD1/39, RIE.

81 Annie Gill, ‘Lecture Notes’, 1907; 1910–1912, LHB1/108/18, RIE, 1.

82 Alexander Hugh Freeland Barbour, ‘Under a Rowan Tree’ (1908, 1925 reprint), LHB1/104/17, RIE, 7–8.

83 Barbour reports that Miss Spencer, Lady Superintendent between 1887 and 1907, similarly believed that the ‘discipline of home life is the best preliminary training’. Barbour, ‘Under a Rowan Tree’, 7.

84 ‘Lectures to Nursing Staff, 1896–1918’, KH/N/FP8/1, KCH Archive, KCL.

85 Pennington, ‘Listerism, its decline and its persistence’’ 48; Worboys, *Spreading Germs*, 166.

86 Russell Howard, *Surgical Nursing and the Principles of Surgery for Nurses* (London: Edward Arnold, 1908), 7; Wood, ‘Supporting or Sabotaging the Surgeon’s Efforts’, 2714.

87 Depending on the examiner, scenario-based questions were common in medical students’ written examinations at least from the early 1870s; for example, at Edinburgh University two questions James Spence set for his examination in Surgery in June 1874 were scenario questions, asking at the end of each, ‘what is the disease and what treatment would you recommend?’ *Edinburgh University Calendar*, 1875–6, 294.

88 Watson, *Handbook for Nurses*, 411–12.

89 Albert Carless, ‘Presentation to Winnipeg Medical Association’, 1911–1936, MS0064/7, Carless Personal Archive, Royal College of Surgeons London.

90 Albert Carless, ‘Lecture notes: Surgery lectures’, 1900–11, KH/PP1, KCL.

91 Rose and Carless, *Manual of Surgery*, 277.

92 One probationer recalled watching him kick a bucket around a lecture theatre after something displeased him: ‘Letters to Miss Oliver’, KH/NL2/1; ‘Unidentified reminiscences’ [1950–80], KH/NL2/3, KCL.

93 For medical students, see Jones, *The Medical Trade Catalogue in Britain.*

94 KCH’s copies of both A. Millicent Ashdown’s *A Complete System of Nursing*, 1917 and Gullan’s 1920 edition of *Theory and Practice of Nursing* contained a stamp from ‘King’s College Hospital Nurses’ Medical Library’.

95 The General Nursing Council’s first ‘syllabus of training’ acknowledged continuing variation: it was an aid, not a set of requirements, for training schools to develop a training ‘scheme by which the candidates may be instructed in the subjects prescribed in detail by the Syllabus of Subjects for Examination’. It also explicitly acknowledged ‘the value of lectures and demonstrations given by Medical Practitioners and others on special subjects on which they are experts’. In particular, ‘bacteriology may be desired as a separate subject, by more advanced schools, but in the submitted scheme it can find place under Elementary Hygiene or Theoretical Nursing … and will be dealt with by both Physician and Surgeon in the senior courses of lectures’. General Nursing Council for England & Wales, *Syllabus of Lectures and Demonstrations for Education and Training in General Nursing* (GNC, 1923), 1–3.

96 ‘Minutes of matron’s and sister’s meetings, c1918–1936’, 23 May 1923, HO1/ST/C1/1, StTH.

97 Wood, ‘Supporting or Sabotaging the Surgeon’s Efforts’, 2714.

